# eMERGEing progress in genomics—the first seven years

**DOI:** 10.3389/fgene.2014.00184

**Published:** 2014-06-17

**Authors:** Dana C. Crawford, David R. Crosslin, Gerard Tromp, Iftikhar J. Kullo, Helena Kuivaniemi, M. Geoffrey Hayes, Joshua C. Denny, William S. Bush, Jonathan L. Haines, Dan M. Roden, Catherine A. McCarty, Gail P. Jarvik, Marylyn D. Ritchie

**Affiliations:** ^1^Center for Human Genetics Research, Vanderbilt UniversityNashville, TN, USA; ^2^Department of Molecular Physiology and Biophysics, Vanderbilt UniversityNashville, TN, USA; ^3^Medical Genetics, Department of Medicine, School of Medicine, University of WashingtonSeattle, WA, USA; ^4^Department of Genome Sciences, University of WashingtonSeattle, WA, USA; ^5^The Sigfried and Janet Weis Center for Research, Geisinger Health SystemDanville, PA, USA; ^6^Division of Cardiovascular Diseases and the Gonda Vascular Center, Mayo ClinicRochester, MN, USA; ^7^Division of Endocrinology, Metabolism, and Molecular Medicine, Department of Medicine, Feinberg School of Medicine, Northwestern UniversityChicago, IL, USA; ^8^Department of Biomedical Informatics, Vanderbilt UniversityNashville, TN, USA; ^9^Department of Medicine, Vanderbilt UniversityNashville, TN, USA; ^10^Department of Epidemiology and Biostatistics, Case Western Reserve UniversityCleveland, OH, USA; ^11^Institute for Computational Biology, Case Western Reserve UniversityCleveland, OH, USA; ^12^Department of Pharmacology, Vanderbilt UniversityNashville, TN, USA; ^13^Essentia Institute of Rural HealthDuluth, MN, USA; ^14^Department of Biochemistry and Molecular Biology, Pennsylvania State UniversityUniversity Park, PA, USA; ^15^Center for Systems Genomics, Pennsylvania State UniversityUniversity Park, PA, USA

**Keywords:** biobanks, genome-wide association studies, pharmacogenomics, electronic medical records

## Abstract

The electronic MEdical Records & GEnomics (eMERGE) network was established in 2007 by the National Human Genome Research Institute (NHGRI) of the National Institutes of Health (NIH) in part to explore the utility of electronic medical records (EMRs) in genome science. The initial focus was on discovery primarily using the genome-wide association paradigm, but more recently, the network has begun evaluating mechanisms to implement new genomic information coupled to clinical decision support into EMRs. Herein, we describe this evolution including the development of the individual and merged eMERGE genomic datasets, the contribution the network has made toward genomic discovery and human health, and the steps taken toward the next generation genotype-phenotype association studies and clinical implementation.

## Introduction

Revolutions in genotyping technology (Ragoussis, 2009) and computational power coupled with the creation of public scientific resources such as The Human Genome Project (2001; Venter et al., 2001), The International HapMap Project (2003; The International HapMap Consortium 2005), and most recently the 1000 Genomes Project (2012), have accelerated genomic discovery, most commonly through genome-wide association studies (GWAS). As of late March 2014, the National Human Genome Research Institute (NHGRI) GWAS catalog listed 1201 publications with 3961 SNPs associated with approximately 571 human diseases and traits at a significance threshold of 5.0 × 10^−8^ (Welter et al., 2014) (https://www.genome.gov/26525384)

The majority of genomic discoveries published to date have been from case-control or cohort epidemiologic studies that collected specific health-related data and DNA samples. These traditional epidemiologic collections already exist and are primed for genomic discovery studies (Willett et al., 2007), making them ideal for large-scale GWAS. Also, although currently under-utilized in genomic discovery, many of the cohorts have collected exposure data that can be interrogated for gene-environment interaction studies (Manolio et al., 2006; Thomas, 2010). However, a major disadvantage of accessing existing epidemiologic cohorts for genomic discoveries is limited representation of diverse racial/ethnic groups (Rosenberg et al., 2010) and of children (Collins and Manolio, 2007). Also, the existing health-related data can be limiting, especially for cohorts or case-controls collections designed with very specific disease outcomes for study such as cancers or cardiovascular disease. Finally, establishing and maintaining an on-going cohort study can pose significant cost burden (Rukovets, 2013).

The disadvantages of accessing existing case-control and cohort studies coupled with the continued need for genotype-phenotype data for genomic discoveries led to the consideration of alternative study designs and data sources such as biorepositories linked to electronic medical records (EMRs). In addition for the potential for large sample sizes of diverse groups, biobanks linked to EMRs make possible the study of many different outcomes and traits, many of which may not be routinely collected by traditional epidemiologic cohorts. And, in this burgeoning era of precision or personalized medicine, biobanks in clinical settings offer unprecedented opportunities to quickly translate research findings to improvements in patient care.

**Figure 1:**
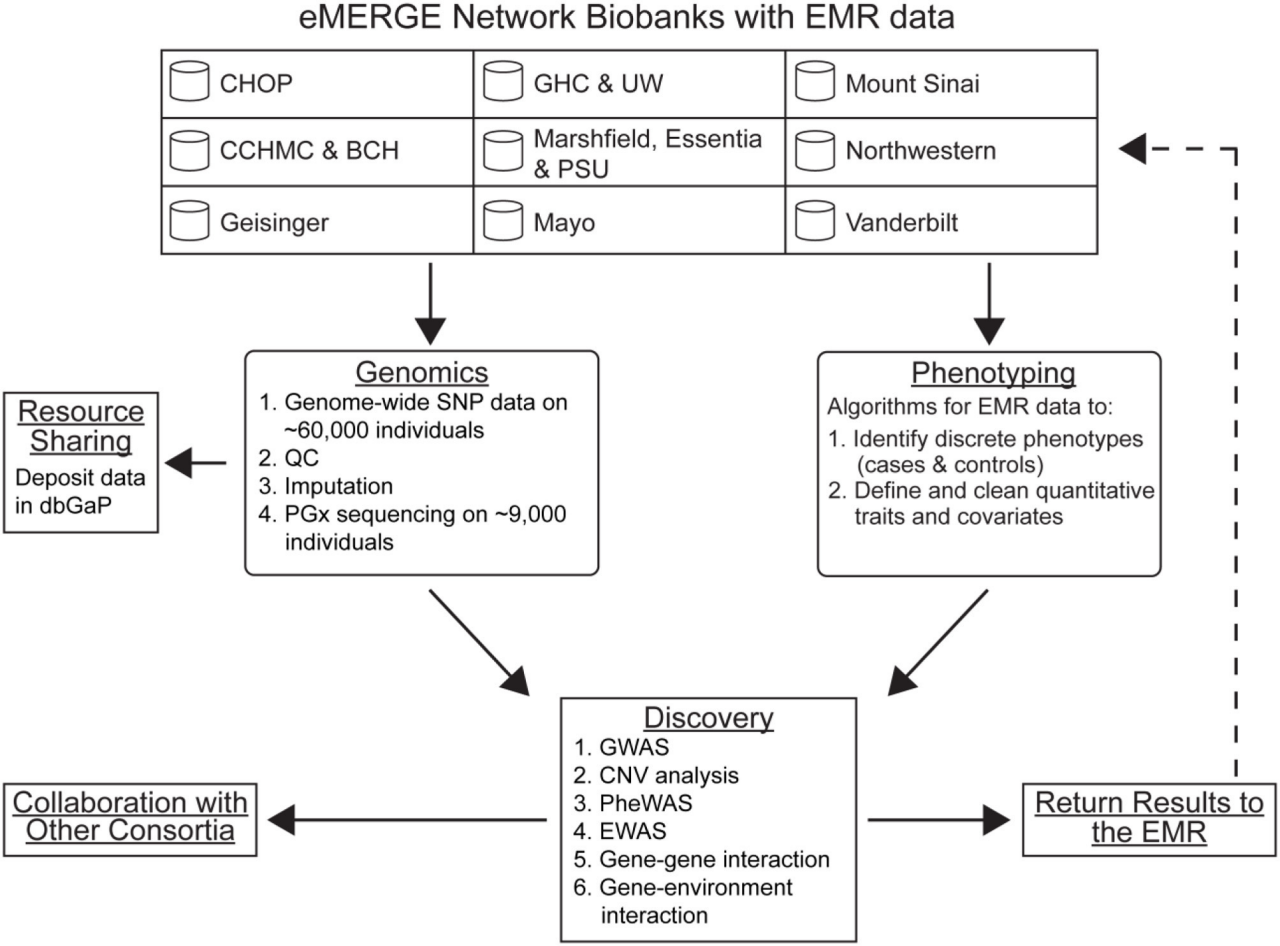
**Major activities of the Genomics Work Group of the eMERGE network**. Abbreviations: CHOP, Children's Hospital of Philadelphia; CCHMC, Cincinnati Children's Hospital Medical Center; BCH, Boston Children's Hospital; GHC, Group Health Cooperative; UW, University of Washington; PSU, Pennsylvania State University; QC, quality control; EMR, electronic medical record; PheWAS, phenomewide association study; EWAS, environment-wide association study; CNV, copy number variation; PGx, pharmacogenomics.

In recognition of the potential for EMR-linked biobanks to genomic discovery and personalized medicine, NHGRI established the electronic MEdical Records & GEnomics (eMERGE) network. The eMERGE network began in 2007 with a Coordinating Center (Vanderbilt University) and five study sites: Group Health/University of Washington, Marshfield Clinic, Mayo Clinic, Northwestern University, and Vanderbilt University (McCarty et al., 2011). The network expanded to include new adult study sites (The Icahn School of Medicine at Mount Sinai and Geisinger Health System) in 2011 as well as pediatric study sites in 2012 (Children's Hospital of Philadelphia and Boston Children's Hospital/Cincinnati Children's Hospital Medical Center) (Gottesman et al., 2013). The major goals of eMERGE I (McCarty et al., 2011) have evolved with experience, and the major activities of the Genomics Work Group of the eMERGE II network are outlined in Figure 1. Here we review from the perspective of the eMERGE Genomics Work Group the contributions the network has made toward genomic discovery since 2007. We also foreshadow the eMERGE network's contributions to the second generation of genotype-phenotype associations as well as implementation of genomic medicine.

## eMERGE genomic resources

The first few years of the eMERGE network required data generation both at the phenotype and genotype levels (McCarty et al., 2011; Gottesman et al., 2013). In the first phase of the eMERGE network, each study site proposed an outcome or trait for phenotype algorithm development and selection of DNA samples for genotyping. Since EMR data are generated for the purposes of clinical care, a necessary step to identifying populations of interest was to create and validate algorithms that queried data elements from the EMR to find phenotypes of interest (Kho et al., 2011; Newton et al., 2013). Typically, these algorithms involved Boolean combinations of billing codes, medication exposures, laboratory, and test results, and/or natural language processing. All algorithms and their validation results in the eMERGE network are available on PheKB (www.phekb.org).

After validation of phenotype algorithms by blinded review, typically by physicians, matching case, and control samples were genotyped. All DNA samples were genotyped using either the Illumina 660-Quad (primarily for participants of European ancestry) or the Illumina 1M (primarily for participants of African ancestry) at either the Broad Institute Center for Genotyping and Analysis or the Center for Inherited Disease Research (CIDR). The eMERGE Coordinating Center established a pipeline to process each study site's data for quality control, data cleaning, and eventual Database of Genotypes and Phenotypes (dbGaP) (Mailman et al., 2007) documentation and deposition (Turner et al., 2011a). The initial round of phenotyping and genotyping resulted in the generation of GWAS-level data on 19,637 samples, of which 18,663 passed quality control metrics. The phenotypes and samples sizes available from these eMERGE phase I efforts included cataracts/HDL-C (2642 cases and 1322 controls; led by Marshfield Clinic), dementia (1241 cases and 2043 controls; led by Group Health Cooperative/University of Washington), electrocardiographic traits (3034 individuals; led by Vanderbilt University), peripheral artery disease (1641 cases and 1604; controls led by Mayo Clinic), and type 2 diabetes (2706 cases and 1496 controls; led by Northwestern University).

During phase I of the eMERGE network, high-density genotyping had matured such that many large cohorts and biorepositories linked to EMRs had existing GWAS-level data. This included expanded genotype datasets at some eMERGE I sites and as such, no new high density genome-wide genotyping was performed in eMERGE phase II. All existing and new study sites in eMERGE II offered existing data on a variety of genotyping platforms and genetic ancestries. With the inclusion of the eMERGE phase I data, a total of 60,766 (47,507 adult and 13,259 pediatric) samples with GWAS-level genotypes or other large-scale data [such as Metabochip (Voight et al., 2012)] generated by either Illumina or Affymetrix arrays are available for study in eMERGE phase II. As detailed in a separate manuscript (Verma et al., in press), pooling and merging of these data required imputation and extensive quality control. The current eMERGE phase II merged dataset (version 2) available for analysis includes 51,038 samples linked to EMRs imputed to >36 million SNPs using the 1000 Genomes Project cosmopolitan reference panel (*n* = 1092) and IMPUTE2 (Verma et al., in press).

New to eMERGE phase II is the eMERGE-PGx project, which involves the targeted sequencing of 84 pharmacogenes identified by the Pharmacogenomics Research Network (PGRN) using DNA capture and contemporary sequencing technologies (known as PGRN-Seq) (Rasmussen-Torvik et al., in press). For this effort, each eMERGE II study site is enrolling ~1000 patients as a pilot study of pharmacogenetic sequencing in clinical practice. Enrollment and sequencing is on-going, and the anticipated network-wide sample size is 9000. All variants annotated through this effort will be available in summary data form via the eMERGE on-line resource “*S*equence, *P*henotype, and p*H*armacogenomics *IN*tegration e*X*change” or “SPHINX” (www.emergesphinx.org). The eMERGE-PGx project will help establish best practices for implementing personalized medicine including exploring and establishing guidelines for returning results to physicians and patients (Kullo et al., 2014). These data will also contribute toward the catalog of rare and less common variants and couple them to EMR data which may increase their clinical utility.

## eMERGE genomic discoveries

It was recognized early in the phenotype and genotype data generation phase of eMERGE I that large sample sizes are needed to have sufficient statistical power for genetic association studies. Indeed, initial GWAS of single eMERGE study site datasets demonstrated that known genotype-phenotype associations such as *SCN10A* and PR duration (Chambers et al., 2010; Holm et al., 2010; Pfeufer et al., 2010) could be replicated albeit at a significance threshold above 5.0 × 10^−8^ (Denny et al., 2010b). While this exercise of replication demonstrated that EMR-derived phenotypes could be used in genotype-phenotype studies, genomic discovery of new associations would require larger sample sizes.

To achieve this goal, the eMERGE network employed several strategies, including (1) pooled analysis across the network, (2) meta-analysis within and with outside consortia, and (3) generation of new phenotype and genotype data for new studies. In the first strategy, each eMERGE study site deployed not only the phenotype used to select study subjects for the genotype-phenotype association studies of the site's primary phenotype, but also the phenotype algorithms designed by other sites to identify additional cases and controls with existing GWAS-level genotyping for these secondary phenotypes, This strategy was successful and identified >15,000 additional samples with existing GWAS-level data to be repurposed for other phenotypes. This effort to share and deploy phenotype algorithms across sites enabled network-wide genomic discoveries for a variety of quantitative traits (Table 1) and facilitated data sharing for meta-analysis efforts outside of the eMERGE network for complex diseases such as late onset Alzheimer's disease (Naj et al., 2011) and electrocardiographic traits (Jeff et al., in press).

**Table 1:** **eMERGE and genomic discovery**.

**Phenotype**	**Nearest gene (rs number)**	**Genetic effect size**	***P***	**Study design (Population)**	**Sample size**	**References**
Alzheimer's Disease, late onset	*BIN1*	*OR* = 1.17	4.2 × 10–14	Consortium meta-analysis, replication	8309 cases	Naj et al., 2011
	(rs7561528)	(95% CI: 1.13, 1.22)		(EA)	7366 controls	
	*CD2AP*	*OR* = 1.11	8.6 × 10–9	Consortium meta-analysis, discovery + replication	18,762 cases	
	(rs9349407)	(95% CI: 1.07, 1.15)		(EA)	29,827 controls	
	*CD33*	*OR* = 0.91	1.6 × 10–9	Consortium meta-analysis, discovery + replication	18,762 cases	
	(rs3865444)	(95% CI: 0.88, 0.93)		(EA)	29,827 controls	
	*CLU*	*OR* = 0.89	1.9 × 10–8	Consortium joint-analysis, replication	8309 cases	
	(rs1532278)	(95% CI: 0.85, 0.93)		(EA)	7366 controls	
	*CR1*	*OR* = 1.16	4.6 × 10–10	Consortium meta-analysis, replication	8309 cases	
	(rs6701713)	(95% CI: 1.11, 1.22)		(EA)	7366 controls	
	*EPHA1*	*OR* = 0.90	6.0 × 10–10	Consortium meta-analysis, discovery + replication	18,762 cases	
	(rs11767557)	(95% CI: 0.86, 0.93)		(EA)	35,597 controls	
	*MS4A4A*	*OR* = 0.88	1.7 × 10–9	Consortium meta-analysis, discovery + replication	8309 cases	
	(rs4938933)	(95% CI: 0.85, 0.92)		(EA)	7366 controls	
	*PICALM*	*OR* = 0.87	7.0 × 10–11	Consortium meta-analysis, replication	8309 cases	
	(rs561655)	(95% CI: 0.84, 0.91)		(EA)	7366 controls	
Erythrocyte sedimentation rate	*C1orf63*	β = −0.09	2 × 10–9	eMERGE joint analysis, discovery + replication	7607 individuals	Kullo et al., 2011
	(rs1043879)			(EA)		
	*CR1*	β = −0.18	3 × 10–26	eMERGE joint analysis, discovery + replication	7607 individuals	
	(rs650877)			(EA)		
	*CRIL*	β = 0.10	2 × 10–9	eMERGE joint analysis, discovery + replication	7607 individuals	
	(rs7527798)			(EA)		
	*TMEM50A*	β = −0.10	2. × 10–13	eMERGE joint analysis, discovery + replication	7607 individuals	
	(rs25547372)			(EA)		
	*TMEM57*	β = −0.10	1 × 10–12	eMERGE joint analysis, discovery + replication	7607 individuals	
	(rs25631242)			(EA)		
	*TMEM57*	β = −0.10	5 × 10–13	eMERGE joint analysis, discovery + replication	7607 individuals	
	(rs25641524)			(EA)		
HDL-C	*CETP*	β = 2.25	1.22 × 10–25	eMERGE analysis, replication	3740 individuals	Turner et al., 2011b
	(rs3764261)	(*SE* = 0.21)		(EA)		
	*LIPC*	β = 2.00	3.92 × 10–14	eMERGE analysis, replication	3740 individuals	
	(rs11855284)	(*SE* = 0.26)		(EA)		
Hypothyroidism	*FOXE1*	*OR* = 0.74	3.96 × 10–9	eMERGE joint analysis, discovery	1317 case	Denny et al., 2011
	(rs7850258)	(95% CI: 0.67, 0.82)		(EA)	5053 controls	
LDL-C	*APOE*	β = −20.0 mg/dl	6.3 × 10–11	eMERGE joint analysis, discovery	618 individuals	Rasmussen-Torvik et al., 2012
	(rs7412)	(95% CI: −25.9, −14.1)		(AA)		
Monocyte count	*CCBP2*	β = 0.32	2.39 × 10–8	eMERGE joint analysis, discovery	11,014 individuals	Crosslin et al., 2013
	(rs2228467)			(EA)		
	*IRF8*	β = −0.25	6.32 × 10–18	eMERGE joint analysis, discovery	11,014 individuals	
	(rs424971)			(EA)		
	*ITGA4*	β = −0.22	1.35 × 10–14	eMERGE joint analysis, replication	11,014 individuals	
	(rs2124440)			(EA)		
	*RPN1*	β = −0.22	4.52 × 10–14	eMERGE joint analysis, replication	11,014 individuals	
	(rs2712381)			(EA)		
PheWAS	*EXOC2*	*OR* = 1.32	1.9 × 10–8	eMERGE pooled analysis, discovery for actinic keratosis	13,835 individuals	Denny et al., 2013
	(rs12210050)	(95% CI: 1.20, 1.45)		(EA)		
	*IRF4*	*OR* = 1.69	4.1 × 10–26	eMERGE pooled analysis, discovery for actinic keratosis	13,835 individuals	
	(rs12203592)	(95% CI: 1.53, 1.86)		(EA)		
	*IRF4*	*OR* = 1.50	3.8 × 10–17	eMERGE pooled analysis, discovery for non-melanoma skin cancer	13,835 individuals	
	(rs12203592)	(95% CI: 1.36, 1.64)		(EA)		
	*NM37*	*OR* = 3.71	2.0 × 10–12	eMERGE pooled analysis, discovery for hypercoagulable state	13,835 individuals	
	(rs16861990)	(95% CI: 2.57, 5.34)		(EA)		
	*TYR*	*OR* = 1.28	2.6 × 10–10	eMERGE pooled analysis, discovery for non-melanoma skin cancer	13,835 individuals	
	(rs1847134)	(95% CI: 1.18, 1.38)		(EA)		
Platelets	*ARHGEF3*	β = −0.19	9.0 × 10–34	eMERGE pooled analysis, discovery for mean platelet volume	6291 individuals	Shameer et al., 2014
	(rs1354034)			(EA)		
	*ARHGEF3*	β = 7.97	6.0 × 10–24	eMERGE pooled analysis, discovery for platelet counts	13,424 individuals	
	(rs1354034)			(EA)		
	*BET1L*	β = −6.46	5.0 × 10–12	eMERGE pooled analysis, discovery for platelet counts	13,424 individuals	
	(rs11602954)			(EA)		
	*DNM3*	β = 0.09	2.0 × 10–8	eMERGE pooled analysis, discovery for mean platelet volume	6291 individuals	
	(rs2180748)			(EA)		
	*FLJ36031-PIK3CG*	β = −0.15	5.0 × 10–22	eMERGE pooled analysis, discovery for mean platelet volume	6291 individuals	
	(rs342240)			(EA)		
	*HBS1L-MYB*	β = −5.42	9.0 × 10–10	eMERGE pooled analysis, discovery for platelet counts	13,424 individuals	
	(rs4895441)			(EA)		
	*JMJD1C*	β = 0.13	3.0 × 10–16	eMERGE pooled analysis, discovery for mean platelet volume	6291 individuals	
	(rs4379723)			(EA)		
	*NFE2*	β = −0.09	2.0 × 10–9	eMERGE pooled analysis, discovery for mean platelet volume	6291 individuals	
	(rs10506328)			(EA)		
	*RCL1*	β = 4.94	1.0 × 10–9	eMERGE pooled analysis, discovery for platelet counts	13,424 individuals	
	*(rs423955)*			(EA)		
	*SH2B3*	β = −5.33	5.0 × 10–11	eMERGE pooled analysis, discovery for platelet counts	13,424 individuals	
	(rs3184504)			(EA)		
	*TAOK1*	β = 0.10	1.0 × 10–10	eMERGE pooled analysis, discovery for mean platelet volume	6291 individuals	
	(rs9900280)			(EA)		
	*TMCC2*	β = 0.11	3.0 × 10–13	eMERGE pooled analysis, discovery for mean platelet volume	6291 individuals	
	(rs9660992)			(EA)		
	*WDR66*	β = −0.31	6.0 × 10–38	eMERGE pooled analysis, discovery for mean platelet volume	6291 individuals	
	(rs7961894)			(EA)		
QRS duration	*SCN5a*	β = −1.0	1.45 × 10–8	eMERGE pooled analysis, replication	5272 individuals	Ritchie et al., 2013
	(rs1805126)			(EA)		
Red blood cell traits	*G6PD*	β = −0.20	4.0 × 10–13	eMERGE pooled analysis, discovery + replication for RBC count	2315 individuals	Ding et al., 2013
	(rs1050828)	(*SE* = 0.03)		(AA)		
	*G6PD*	β = 2.46	1.0 × 10–14	eMERGE pooled analysis, discovery + replication for mean corpuscular volume	2315 individuals	
	(rs1050828)	(*SE* = 0.32)		(AA)		
	*G6PD*	β = 0.72	9.0 × 10–9	eMERGE pooled analysis, discovery + replication for mean corpuscular hemoglobin	2315 individuals	
	(rs1050828)	(*SE* = 0.12)		(AA)		
	*ITFG3*	β = −3.57	5.0 × 10–29	eMERGE pooled analysis, discovery + replication for mean cell volume	2315 individuals	
	(rs9924561)	(*SE* = 0.32)		(AA)		
	*ITFG3*	β = −1.56 (*SE* = 0.12)	8.0 × 10–36	eMERGE pooled analysis, discovery + replication for mean corpuscular hemoglobin	2315 individuals	
	(rs9924561)	(*SE* = 0.12)		(AA)		
	*ITFG3*	β = −0.47	4.0 × 10–13	eMERGE pooled analysis, discovery + replication for mean corpuscular hemoglobin concentration	2315 individuals	
	(rs9924561)	(*SE* = 0.06)		(AA)		
	(rs7120391)	β = 0.30	5.0 × 10–9	eMERGE pooled analysis, discovery + replication for mean corpuscular hemoglobin concentration	2315 individuals	
		(*SE* = 0.05)		(AA)		
Red blood cell traits	*CDT1*	-0.06	2.0 × 10–8	eMERGE pooled analysis, discovery + replication for mean corpuscular hemoglobin concentration	12,486 individuals	Ding et al., 2012
	(rs837763)			(EA)		
	*PTPLAD1/C15orf44*	0.13	8.0 × 10–9	eMERGE pooled analysis, discovery + replication for mean corpuscular hemoglobin	12,486 individuals	
	(rs8035639)			(EA)		
	*THRB*	0.35	6.0 × 10–9	eMERGE pooled analysis, discovery + replication for mean corpuscular volume	12,486 individuals	
	(rs9310736)			(EA)		
	(rs9937239)	0.06	2.0 × 10–8	eMERGE pooled analysis, discovery + replication for mean corpuscular hemoglobin concentration	12,486 individuals	
				(EA)	
Type 2 diabetes	*TCF7L2*	*OR* = 1.41	2.98 × 10–10	eMERGE meta-analysis, replication	2413 cases	Kho et al., 2012
	(rs7903146)			(EA)	2392 controls	
White blood cell count	*DARC*	β = 1.28	4.92 × 10–24	eMERGE joint analysis, discovery	361 individuals	Crosslin et al., 2012
	(rs12075)	(*SE* = 0.12)		(AA)		
White blood cell count	*GSDMA*	β = 0.14	1.75 × 10–12	eMERGE joint analysis, discovery	13,562 individuals	Crosslin et al., 2012
	(rs3859192)	(*SE* = 0.02)		(EA)		
	*MED24*	β = −0.13	4.92 × 10–10	eMERGE joint analysis, discovery	13,562 individuals	
	(rs9916158)	(*SE* = 0.02)		(EA)		
	*PSMD3*	β = 0.14	3.47 × 10–11	eMERGE joint analysis, discovery	13,562 individuals	
	(rs4065321)	(*SE* = 0.02)		(EA)		

The eMERGE network has conducted or contributed data toward genome-wide association studies. For each study with genome-wide significant results (p < 5 × 10^−8^), we list the primary phenotype, the nearest genes associated, the index rs number, the reported genetic effect size, the p-value, the study design, the population, the sample size, and the reference. Abbreviations: AA, African American; EA, European American; β, beta; CI, confidence interval; OR, odds ratio; SE, standard error.

Implicit in the eMERGE data sharing strategy is the concept that phenotype algorithms are portable across different study sites with different EMRs software systems as well as different health care practices and cultures (Kho et al., 2011). Also, it was assumed that each study site could reuse data collected for a specific phenotype or trait to conduct studies for other unrelated phenotypes without introducing substantial biases. For example, in the type 2 diabetes (T2D) association study, there was considerable heterogeneity in the proportion of type 2 diabetes cases at each site, as well the odds ratio estimates for the index T2D SNP within each site's cohort, but when combined across the sites the odds ratio was indistinguishable from those using larger purposely-collected T2D case-control collections (Kho et al., 2012). These data suggest that potential study heterogeneity was magnified or measurable at the single study level but dampened at the larger network-wide level of analysis.

To further test the boundaries of these assumptions and early observations, eMERGE undertook a network-wide study of hypothyroidism, a new phenotype not related to any of the study site-specific phenotypes. The phenotype algorithm was developed at the Vanderbilt University study site and deployed and evaluated by all eMERGE study sites, like other eMERGE phenotypes. Despite potential differences in billing and coding practices across study sites, a total of 1317 cases and 5053 controls were identified with average weighted positive predictive values of 92.4 and 98.5, respectively (Denny et al., 2011). The subsequent GWAS identified common genetic variants near *FOXE1* associated with European American cases, and the findings were replicated in an independent dataset from the Mayo Genome Consortia as well as externally in the literature (Eriksson et al., 2012). These studies illustrate that existing genotype data linked to EMR data can be reused for other genomic discovery studies, a potentially cost-effective strategy. However, further study is needed to determine the extent of biases that were introduced in the generation of these data that may impact the widespread adoption of this strategy across a range of phenotypes available in the EMR.

As evident in the *FOXE1/*hypothyroidism example, existing genotype data linked to EMR data enable the relatively rapid identification of cases and controls for traditional GWAS where one disease or trait is studied. These data have also enabled the study of pleiotropy, whereby a genetic variant influences or impacts multiple phenotypes or traits (Stearns, 2010; Solovieff et al., 2013). In one popular approach, known as phenome-wide association studies or PheWAS, a GWAS-identified variant is interrogated for other associations throughout the available phenome. PheWAS has been performed in both epidemiologic (Pendergrass et al., 2013a) and EMR-based datasets such as eMERGE (Denny et al., 2010a, 2013). Collectively, these and other data (Sivakumaran et al., 2011) suggest that pleiotropy among GWAS-identified variants is not uncommon. PheWAS conducted in the EMR setting can reveal novel genotype-phenotype pleiotropic relationships not possible in traditional epidemiologic cohorts. For example, a recent PheWAS in the eMERGE participants of European ancestry revealed a potential association between actinic keratosis and *IRF4* rs12203592 (Denny et al., 2013) (Table 1), a GWAS-identified variant previously associated with hair color, eye color, and non-melanoma skin cancer (Han et al., 2008; Eriksson et al., 2010; Zhang et al., 2013).

Much like its contributions toward the study of pleiotropy, the eMERGE network is beginning to make substantial contributions to understudied or burgeoning areas of interest in genomic discovery such as the study of pediatric populations and diverse racial/ethnic groups. Indeed, with the addition of the pediatric study sites, eMERGE II boasts one of the largest collections of pediatric DNA samples linked to EMRs for genomic discovery (Gottesman et al., 2013). The current version (2) of the merged, imputed eMERGE II dataset includes >12,000 pediatric samples linked to EMRs. As of March 15, 2014, fewer than 5% of the GWAS annotated by the NHGRI GWAS Catalog (Welter et al., 2014) mention children as a study population, highlighting the tremendous opportunity for genomic discovery in this cohort. To calibrate the eMERGE II datasets, a site-specific investigation was recently performed for body mass index (BMI) *z*-scores using BMI extracted from the pediatric EMRs and calculated using the Centers for Disease Control and Prevention (CDC) growth charts (Namjou et al., 2013). Similar to epidemiologic datasets (Frayling et al., 2007; Meyre et al., 2009; Scherag et al., 2010), this EMR-based study demonstrated that adult GWAS-identified obesity variants such as those in *FTO* were also relevant for children of European-descent (Namjou et al., 2013). Genomic discovery using GWAS in pediatric populations is currently underway in eMERGE II for complex phenotypes such as autism and asthma.

In the past several years, most GWAS have included individuals of European ancestry (Rosenberg et al., 2010). Indeed, only approximately 10% of the GWAS annotated in the NHGRI GWAS Catalog include populations of African ancestry (https://www.genome.gov/26525384). The eMERGE network is significantly poised to contribute to GWA studies for populations of non-European ancestry given that several study sites (notably Northwestern University, Vanderbilt University, and The Icahn School of Medicine at Mount Sinai) include participants of African ancestry. eMERGE I has already contributed genome-wide associated variants (at a threshold of *p* < 10^−5^) in participants of African ancestry to the NHGRI GWAS Catalog for LDL-C (Rasmussen-Torvik et al., 2012), red blood cell traits (Ding et al., 2013), white blood cell traits (Crosslin et al., 2012), type 2 diabetes (Kho et al., 2012), and electrocardiographic traits (Jeff et al., 2013). As an extension of GWAS, eMERGE investigators have also begun fine-mapping GWAS-identified regions to identify the best index variant in African ancestry populations as well as exploring alternative genomic discovery methods such as admixture mapping to identify potentially novel or population-specific associations (Jeff et al., 2014).

Beyond conventional GWAS, the eMERGE network has also led efforts to identify genetic (G × G) and environmental (G × E) modifiers of common, complex phenotypes. In an early example, eMERGE investigators used extrinsic biological knowledge via the Biofilter algorithm (Bush et al., 2009) to prioritize genetic variants for SNP-SNP modeling to identify gene-gene interactions relevant for HDL-C (Turner et al., 2011b). The extrinsic biological knowledge approach has also been recently implemented for both G × G and G × E tests of association for cataracts, with the latter including only environmental variables known to be associated with the eye disease (Pendergrass et al., 2013b,c). Finally, eMERGE investigators have implemented environmental-wide association studies (EWAS) to identify and prioritize environmental factors important for type 2 diabetes (Hall et al., 2014), a relatively new approach to identify all possible environmental variables that may be relevant for G × E studies for the disease of interest.

## eMERGE second generation gwas

The majority of GWAS described to date for the eMERGE network represent data and efforts from phase I of the network's existence. Phase II analyses of larger, more diverse sample sizes are on-going (Gottesman et al., 2013). As documented and described in an accompanying article (Verma et al., in press), eMERGE II network datasets include single site datasets, a network-wide merged genotyped dataset, single site imputed datasets, and a network-wide merged imputed dataset; the merged set includes >36 million SNPs for samples from >50,000 individuals linked to EMRs. Imputation of the X-chromosome is underway, and future eMERGE II analyses will include this chromosome. Network-wide efforts are also underway to annotate copy number variants (Connolly et al., 2014) as well as to annotate and identify potentially deleterious null variants. Site-specific efforts are also underway to collect or extract additional standardized environmental data for GxE studies using the PhenX Toolkit (Hamilton et al., 2011; McCarty et al., 2014). Efforts are underway to develop analytical approaches for repeated measures data characteristic of the EMR, to conduct mapping studies for populations with three-way admixture events, and to incorporate phenotyping uncertainty when balancing sample size/power and misclassification (McDavid et al., 2013). With >36 million SNPs, large sample sizes, and phenotypically dense EMRs, eMERGE II and beyond promises to continue genomic discovery in the second generation of GWAS.

### Conflict of interest statement

The authors declare that the research was conducted in the absence of any commercial or financial relationships that could be construed as a potential conflict of interest.
